# Gut-Derived Protein-Bound Uremic Toxins

**DOI:** 10.3390/toxins12090590

**Published:** 2020-09-11

**Authors:** Amanda L. Graboski, Matthew R. Redinbo

**Affiliations:** 1 Department of Pharmacology, University of North Carolina, Chapel Hill, NC 27599-7365, USA; amanda_graboski@med.unc.edu; 2 Departments of Chemistry, Biochemistry, Microbiology and Genomics, University of North Carolina, Chapel Hill, NC 27599-3290, USA

**Keywords:** protein-bound uremic toxins, intestinal microbiota, gut-kidney axis

## Abstract

Chronic kidney disease (CKD) afflicts more than 500 million people worldwide and is one of the fastest growing global causes of mortality. When glomerular filtration rate begins to fall, uremic toxins accumulate in the serum and significantly increase the risk of death from cardiovascular disease and other causes. Several of the most harmful uremic toxins are produced by the gut microbiota. Furthermore, many such toxins are protein-bound and are therefore recalcitrant to removal by dialysis. We review the derivation and pathological mechanisms of gut-derived, protein-bound uremic toxins (PBUTs). We further outline the emerging relationship between kidney disease and gut dysbiosis, including the bacterial taxa altered, the regulation of microbial uremic toxin-producing genes, and their downstream physiological and neurological consequences. Finally, we discuss gut-targeted therapeutic strategies employed to reduce PBUTs. We conclude that targeting the gut microbiota is a promising approach for the treatment of CKD by blocking the serum accumulation of PBUTs that cannot be eliminated by dialysis.

## 1. Introduction

It has been estimated that 500 million people in the world suffer from chronic kidney disease (CKD) [1,2]. The progression of CKD is marked by the gradual loss of the kidneys’ regulatory and filtration capabilities, leaving patients with numerous imbalances and the serum retention of toxic compounds that cause uremic syndrome [3]. The accumulation of uremic toxins has a broad impact on human physiology and is associated with the development of cardiovascular disease, renal fibrosis, neurotoxicity, disrupted hepatic metabolism, and altered bone architecture [4]. Uremic toxins are categorized as (1) small water-soluble molecules, (2) protein-bound compounds, or (3) middle molecules. Although toxins within all three categories have deleterious effects on the body, the most troublesome and hazardous are those that are difficult to remove by dialysis: protein-bound uremic toxins (PBUTs) [5]. Furthermore, many PBUTs derive from the gut as products of the microbial metabolism of dietary compounds [6,7].

Twenty-five gut-derived, protein-bound uremic toxins have been described to date. They can be divided into six primary categories: advanced glycation end-products, hippurates, indoles, phenols, polyamines, and other (Table 1). Protein-bound toxins pose a unique problem in patients suffering from end-stage renal disease (ESRD) as the most effective techniques for removing uremic toxins—dialysis and hemofiltration—are unsuccessful against these molecules. Because PBUTs are not free floating in circulation, only a small fraction of unbound solute is susceptible to the concentration and pressure gradients used to draw waste out of the blood [8]. No therapeutic or other techniques are available to reduce serum levels of gut-derived PBUTs. Therefore, patients have no options to combat uremic syndrome caused by PBUTs, and the subsequent progression from CKD into ESRD.

Gut microbial dysbiosis has been implicated in a number of disorders, including CKD, obesity, inflammatory bowel diseases, type 2 diabetes, and cardiovascular disease [9]. With respect to CKD, there is a well-established relationship between the decline in kidney function and alterations to the gut microbiota [10,11,12,13,14,15,16]. The association between kidney disease and changes in the composition of the gut microflora, intestinal environment, and permeability of the gut epithelial barrier occurs via what has been termed the gut–kidney axis [17,18,19]. Toxic products generated by a dysbiotic gut may contribute to the progression of chronic kidney disease and its numerous comorbidities.

Here we review gut-derived, protein-bound uremic toxins, separating them into two categories: (1) diet-ingested toxins and (2) toxins generated by gut microbial metabolism. Both PBUT categories impact gut microflora composition, and the intake, intestinal absorption, and serum levels of these toxins are altered with each progressing stage of kidney disease [20]. We outline the derivation and pathological mechanisms of each class of gut-derived PBUT, as well as their relationship to the gut microbiota, and we present recent approaches to reduce PBUT levels by targeting the gut microbiome.

## 2. PBUT Derivation and Pathological Mechanisms

### 2.1. Advanced Glycation End Products (AGEs)

**Figure 1:**
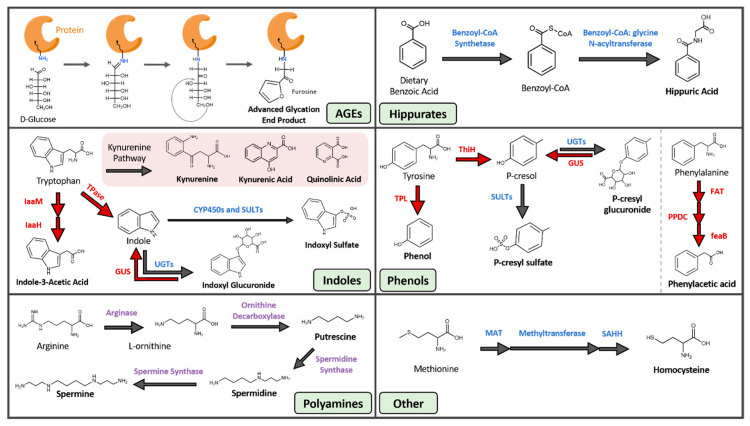
Gut-Derived Protein-Bound Uremic Toxins. Blue text: human enzymes, red text: microbial enzymes, purple text: both human and microbial enzymes. AGE, advanced glycation end product; CYP450, cytochrome P450; FAT, phenylalanine dehydrogenase or transaminase; feaB, phenylacetaldehyde dehydrogenase; GUS, beta-glucuronidase; IaaH, indole-3-acetamide hydrolase; IaaM, tryptophan 2-monooxygenase; MAT, methionine adenosyltransferase; PPDC, phenylpyruvate decarboxylase; SAHH, s-adenosylhomocysteine hydrolase; SULT, sulfotransferase; ThiH, tyrosine lyase; TPase, tryptophanase; TPL, tyrosine phenol-lyase; UGT, UDP-glucuronosyltransferase.

Advanced glycation end products are a heterogenous group of compounds formed through the non-enzymatic Maillard sequence of reactions in which reducing sugars are covalently linked to protein amine groups, most commonly lysine and arginine residues [21] (Figure 1, Table 1). AGEs were traditionally linked to diabetes and hyperglycemia, although more recent studies show that AGEs can form in disease states with high oxidative stress like CKD, even in the absence of hyperglycemia [22]. AGE precursors also originate from smoking. A contemporary diet, especially when cooked under high heat and dry conditions, can significantly contribute to the AGE pool within the body [23]. The modern western diet is filled with heat-treated and pre-treated foods such as cereal, bakery products, and powdered milk that increase systemic AGE levels. Furthermore, numerous studies suggest that increased AGE intake leads to oxidative stress and inflammation [24]. Examples of circulatory AGE precursors are fructoselysine, methylglyoxal, glyoxal, and 3-deoxyglucosone, and most such precursors are products of varying metabolic and oxidative processes such as glycolysis, lipid peroxidation, and the degradation of glycolytic intermediates [25]. Furthermore, the post-translational linkage of glycans to amino acid residues can exert damaging effects on proteins by altering their functional domains, rendering them dysfunctional or inactive [26].

AGEs exert their most toxic effects by binding to and signaling through the advanced glycan end product-specific receptor (RAGE). This multi-ligand receptor is either found on cell membrane surfaces (the full-length receptor) or is secreted by cells (the truncated soluble isoform). RAGE is expressed in a wide range of tissues, but is most concentrated in the heart, lungs, and skeletal muscles [27]. The full-length RAGE signals through NF-κB, mitogen-activated protein kinases, and Jun N-terminal kinase pathways, initiating transcription of proinflammatory cytokines and adhesion molecules leading to inflammation and oxidative stress [28,29]. However, soluble RAGE acts as a decoy and sequesters free floating AGEs [30]. Both full-length and truncated soluble isoforms are upregulated in diseases such as diabetes and autoimmune/inflammatory disorders. Their serum concentrations are correlated with cardiovascular risk factors and disease [21,31]. The actions of AGE-RAGE signaling impair endothelial progenitor cell survival, differentiation, and migration, and thereby induce endothelial dysfunction, which is an early marker of atherosclerosis and arterial stiffness [32,33]. The accumulation of AGEs on proteins within the extracellular matrix can form cross-links, especially on type 1 collagen and elastin, trapping macromolecules as well as increasing the surface area and stiffness of the vasculature [34]. In summary, AGEs lead to arterial stiffness, diabetic nephropathy, endothelial dysfunction, and dysregulation of the immune system [34,35,36].

### 2.2. Hippurates

The hippurates encompass hippuric acid (HA) and hydroxyhippuric acid (Figure 1, Table 1). These molecules are acyl-glycine products formed through the conjugation of benzoic acid and glycine by benzoyl-CoA synthetase and glycine N-acyltransferases [37]. This two-step reaction occurs within the mitochondrial matrix of hepatocytes. Benzoic acid is first converted into benzoyl-CoA by benzoyl-CoA synthetase, and benzyl-CoA is then joined with glycine by benzoyl CoA: glycine N-acetyltransferase to form hippurate [38].

Both HA and hydroxyhippuric acid are normally found in the urine and are commonly used as a measure of renal clearance. Their bodily concentrations are increased with consumption of phenolic compounds, such as those found in wine, fruit, and tea [37,39]. Phenolic compounds can also be generated through aromatic amino acid metabolism by the gut microflora [40]. Regardless of their source, phenolic precursors are converted into benzoic acid that is then utilized by hepatic enzymes to form hippurates. Bodily HA levels can also be elevated due to environmental exposure to toluene. Factory workers and those exposed to high levels of air pollution have elevated HA levels, and for many years this was believed to be a reliable measure of toluene exposure [41,42]. However, there are now doubts about the validity of using HA as a biomarker for toluene exposure due to inter- and intra-individual variabilities caused by diet, medical treatments, and alcohol consumption [43].

Hippurates can induce free radical production in the renal tubule through interactions with organic ion transporters. Free radical production causes cellular toxicity and reduced cellular proliferation [44]. HA also elevates systolic blood pressure and thereby alters hemodynamics and reduces glomerular filtration rate. Additionally, HA inflicts proximal tubule cell damage by inducing the production of fibrotic proteins through NF-κB signaling. Subsequent interstitial fibrosis occurs on the proximal tubule, advancing the progression of chronic kidney disease [44,45]. HA is implicated in upregulation of intracellular adhesion molecule expression and the activation of Dynamin-related protein 1 (Drp1)-mediated mitochondrial fission in vitro and in vivo. This ultimately causes endothelial dysfunction and impaired endothelium-dependent vasodilation [46]. Lastly, HA binds to human serum albumin (HSA) and inhibits the interaction of HSA with other organic and inorganic molecules, altering their pharmacokinetics resulting in toxic consequences [47].

### 2.3. Indoles

Although some of the compounds within this category do not contain an indole ring (e.g., kynurenine, kynurenic acid, and quinolinic acid), we are grouping these toxins under indoles based on similarities in their derivation pathways. The indoles are composed of seven distinct uremic toxins, all of which arise from tryptophan metabolism (Figure 1, Table 1). Tryptophan, which must be obtained through dietary means, is processed through the host serotonin and kynurenine pathways, as well as by several microbial metabolic processes [48]. The products of these pathways impact sleep, mood, and regulatory processes in the body [49]. Tryptophan metabolism is shifted away from serotonin and towards kynurenine production under conditions of infection, stress, and alterations to the gut microbiota [50,51]. The kynurenine cascade creates compounds like kynurenic and quinolinic acid that exert a wide range of effects, including acting as antioxidants, immunosuppressants, neurotoxins, and impacting cellular energy generation [52]. Additionally, the gut microbiota can metabolize tryptophan into indican, indole, indole acid, skatole, and tryptamine derivatives [53]. The microbial pathways that create indole and indole acid are of interest because they are responsible for the production of the uremic toxins indoxyl sulfate (IS), indoxyl glucuronide, and indole-3-acetic acid (IAA). IAA is generated by bacterial tryptophan monooxygenases and indole-3-acetamide hydrolases [54]. IS arises from both microbial and host reactions. Gut microbial tryptophanase enzymes first convert tryptophan into indole, and indole is then trafficked to the liver where it is hydroxylated and sulfated by human hepatic cytochrome P450 and sulfotransferase enzymes, respectively, to the circulating and damaging uremic toxin IS [55].

Many tryptophan-derived uremic toxins, including IAA, IS, kynurenine, and indoxyl glucuronide, activate the aryl hydrocarbon receptor (AhR), which is a ligand-activated transcription factor that mediates toxic and inflammatory responses [56,57,58]. Activated AhR increases the expression of proinflammatory and oxidative stress cell markers such as vascular cell-1 (VCAM-1) and intercellular adhesion molecule-1 (ICAM-1) [59]. Kynurenine, kynurenic acid, IAA, and IS also impact skeletal muscle mitochondrial energetics by disrupting the electron transport chain and associated dehydrogenases. This impairs mitochondrial oxidative phosphorylation and respiratory capacity, and leads to muscle weakness, atrophy, and fatigue [60]. Quinolinic acid acts as an N-methyl-D-aspartate (NMDA) receptor agonist to induce neuronal excitotoxicity [50], and also causes neurotoxicity through inducing excessive glutamate release and lipid peroxidation [61].

IS is the most notable uremic toxin of the indole class due to its cardiovascular and renal implications. IS induces pro-fibrotic, pro-inflammatory, and pro-hypertrophic effects through NF-κB and mitogen-activated protein kinases pathways in vitro and in vivo [62]. IS also causes endothelial dysfunction, a prothrombotic state, and impairs neovascularization, all of which promote the development of cardiovascular disorders [63]. Indeed, the pro-inflammatory properties of IS are associated with at least six phenotypes of cardiovascular disease (CVD), including arrhythmia [64,65], arteriosclerosis [66], atherosclerosis [67,68], congestive heart failure [62], peripheral artery disease [69], and vascular access thrombosis [70]. IS is also implicated in bone diseases through its observed reduction of parathyroid hormone (PTH) expression in rodent osteoblasts and in its reduction of bone turnover in rats fed high indole diets [71,72]. Numerous pre-clinical studies in various cell lines and animal models show strong connections between IS toxicity and negative impacts on the cardiovascular, renal, and skeletal systems [73].

### 2.4. Phenols

A range of phenolic compounds are generated from the gut microbial fermentation of the dietary amino acids tyrosine and phenylalanine (Figure 1, Table 1). The uremic toxins phenol, hydroquinone, *p*-cresyl sulfate (pCS), and *p*-cresyl glucuronide all derive from the microbial metabolism of tyrosine, while phenylacetic acid is generated from the microbial breakdown of phenylalanine [74,75,76]. Hydroquinone arises both from cigarette smoke and the microbial metabolism of dietary amino acids. While hydroquinone exerts tumorigenic effects in mice by inducing tubule-cell carcinoma [77] and is mutagenic in rodent bone-marrow cells in vitro, evidence of hydroquinone genotoxicity in humans is limited [8]. In contrast, the toxicity of phenylacetic acid is well understood. Phenylacetic acid inhibits nitric oxide synthase (iNOS) expression, which leads to reduced nitric oxide production and reduced protection against atherogenesis and inflammation in vessel walls [78]. Phenylacetic acid also enhances the production of ROS in vascular smooth muscle cells and increases inflammatory response by polymorphonuclear leukocytes [79,80]. Together, these effects produce a highly oxidative environment and systemic inflammation, two defining features of CKD.

The most well-studied and damaging phenolic uremic toxin is pCS, which is produced via multiple steps involving both gut microbial and host hepatic factors. Gut microbial enzymes can convert tyrosine into *p*-cresol directly by tyrosine lyases (ThiH) or through a multi-step process involving tyrosine transaminases and 4-hydroxylphenylacetate decarboxylases [81]. The majority of gut-generated *p*-cresol is absorbed into systemic circulation, while a small fraction remains in the gut and is converted into *p*-cresyl glucuronide by host epithelial UDP-glucuronosyltransferases. The *p*-cresol that reaches systemic circulation is converted by hepatic sulfotransferases into the uremic toxin pCS [82].

Increased levels of pCS in human serum and urine have been repeatedly associated with cardiovascular and all-cause mortalities in patients, regardless of dialysis treatment [83]. In rats, pCS causes cardiac toxicity and dysfunction by NADPH oxidase activation and ROS production, which induce cardiomyocyte apoptosis and diastolic dysfunction [84]. pCS also stimulates endothelial microparticle release, a sign of endothelial damage, as well as oxidative stress in vascular smooth muscle cells. The release of microparticles decreases both endothelial repair and the formation of new vessels as well as increases the senescence of mature endothelial cells [85]. pCS further causes inward eutrophic vascular remodeling and contractions of the aorta by direct stimulation of Rho-associated protein kinase, which is known to play a role in regulating cellular shape and movement [86]. In addition to the induction of cardiovascular damage, pCS has been implicated in the progression of renal damage [87]. It activates the renin angiotensin aldosterone system and induces epithelial-to-mesenchymal transitions, both of which contribute to fibrosis and advance kidney injury [88]. The alternative product of *p*-cresol, *p*-cresyl glucuronide, is present in lower concentrations but is also correlated with all-cause mortality independent of pCS levels [89]. *P*-cresyl glucuronide appears to upregulate breast cancer resistance protein (BCRP) activity and induce stress in renal tubule cells [87,90].

### 2.5. Polyamines

Polyamines in this context refer to three small aliphatic amines: putrescine, spermidine, and spermine (Figure 1, Table 1). All three molecules arise from a range of sources including gut microbial metabolism, host biosynthesis, and dietary intake. Polyamines produced by gut microbial metabolism derive from the breakdown of arginine. Arginase converts arginine into urea and L-ornithine, which is further metabolized by ornithine decarboxylase into polyamines [91]. Both gut microbial and human cells synthesize polyamines [92], with mammalian cells employing spermidine and spermine in metabolism, ion channel function, nucleic acid packaging, and DNA replication [93]. Similar to microbes, host cells synthesize these polyamines from arginine and its metabolite L-ornithine. Finally, almost all foods contain polyamines, and thus aliphatic amines can arise directly from the diet and are particularly abundant in soybeans, mushrooms, beef, pork, and green tea leaves [94].

The uremic toxicity of spermidine and spermine is controversial because studies have shown lower serum levels of these toxins in CKD patients compared that of healthy controls [95,96]. Higher serum levels in CKD is one of five requirements for classification as a uremic toxin. The same investigations, however, revealed increased levels of putrescine in CKD patients. A confounding factor in these measurements is the fact that polyamines are primarily located intracellularly; thus, serum levels may not accurately reflect total concentration or accumulation. Polyamines inhibit the activity of erythropoietin, which may reduce the proliferation of erythroid precursor cells that are directly involved in anemia, a staple in chronic renal failure patients [97]. At concentrations higher than those observed in uremia, spermidine and spermine kill aortic endothelial cells and rat aortic smooth muscle cells [98]. While evidence supporting the direct toxicity of polyamines is limited, there is ample proof that the intermediates of polyamine metabolism are toxic. Spermidine and spermine are metabolized into hydrogen peroxide, ammonium, and acrolein, all of which are cellular toxins [99]. In animal models of CKD, acrolein induced dyslipidemia and cardiac damage [100], and acrolein in concentrations as low as 5 µM inhibited cell culture growth by 50% [101]. It should also be noted that serum levels of acrolein are elevated in CKD patients and it has been suggested that acrolein should formally be considered a uremic toxin [96,102].

### 2.6. Other

The two-remaining gut-derived PBUTs are 3-carboxy-4-methyl-5-propyl-2-furanpropionic acid (CMPF) and homocysteine (Figure 1, Table 1). CMPF is a major endogenous metabolite of furan fatty acid metabolism and is often incorporated into phospholipids and cholesterol esters [103]. The richest sources of CMPF are dietary fish and fish oils [104], while homocysteine arises from dietary land animal proteins. The sulfhydryl-containing amino acid homocysteine is produced from the multistep demethylation of dietary methionine [105,106].

CMPF is essentially 100% protein bound [107] and impacts the elimination of bilirubin [108] and thyroxine [109]. Like HA and IS, CMPF can compete with drugs, metabolites, and endogenously produced organic acids for renal excretion by organic anion transporters (OATs) [110]. OAT disruption can also alter the transport of organic anions at the blood brain barrier, leading to neurological abnormalities. Indeed, CMPF serum levels were correlated with neurological abnormalities in one clinical study [111]. In addition to altering drug and metabolite pharmacokinetics, CMPF impacts a number of liver metabolic processes, including inhibition of hepatic glutathione-S-transferase [112], transport and deiodination of thyroid hormone T4 [113], and the hepatic uptake of drugs and xenobiotics [114]. Finally, CMPF accumulates at high concentrations in renal tubule cells and interacts with oxygen free radicals to form CMPF radical adducts. These adducts then engage dissolved oxygen to produce more oxygen free radicals, leading to the production of TGF-beta and cellular damage [115]. Together, these processes in renal tubule cells accelerate renal damage and advance the progression of CKD.

Homocysteine stimulates the proliferation of vascular smooth muscle cells and reduces the growth of endothelial cells, both of which are hallmarks of atherosclerosis [116]. Homocysteine inhibits the expression of thrombomodulin and the binding of tissue plasminogen activator to endothelial cells, interfering with fibrinolytic and thrombotic functions [117]. A rodent study demonstrated that homocysteine stimulates the expression of inflammatory and oxidative stress markers in the gut, including TNF-alpha and IL-6, leading to the degradation of tight-junctions and increased intestinal permeability [118]. The role of homocysteine in poor cardiovascular outcomes in CKD patients is controversial because randomized controlled trials and observational studies have provided conflicting results [119,120]. However, homocysteine levels are dependent on several factors, including diet, vitamin intake, and genetic predispositions, which may confound the clinical connection between homocysteine and cardiovascular outcomes.

## 3. PBUTs and the Gut Microbiome

### 3.1. Gut Microbial Dysbiosis

The uremic nature of CKD has a profound impact on the intestinal microbiota. The composition of the gastrointestinal microflora is significantly altered in ESRD [10,11,12], and is impacted even in the early stages of kidney disease [13]. Stool samples of healthy controls and ESRD patients exhibited significant differences in 190 bacterial taxa belonging to 23 different families. *Lactobacillaceae* and *Prevotellaceae* were lower in ESRD, whereas *Enterobacteria* and *Enterococci* taxa increased in colonic abundance by 100-fold [10]. Controlled studies utilizing uremic rats show renal dysfunction itself induces alterations in the composition of the gut microbiota, identifying 175 bacterial OTUs that differed between uremic and control animals [10].

Patients with kidney disease experience intestinal wall edema and congestion, slower transit times, metabolic acidosis, and decreased processing of dietary fibers. Each of these factors alters gut epithelial tight-junctions, affects the translocation of microbial metabolites, and increases intestinal permeability [17]. Together, they impair immune system function and lead to systemic inflammation, which furthers gut dysbiosis and advances kidney damage.

**Figure 2:**
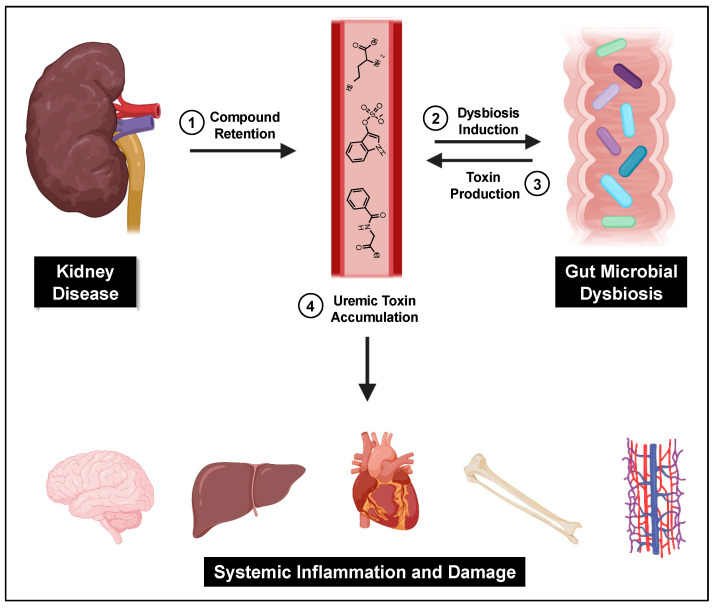
The gut–kidney axis. Impaired kidney function and other CKD-related causes lead to uremic toxin accumulation and gut dysbiosis, which then furthers gut-derived uremic toxin accumulation and subsequent systemic damage.

A central cause proposed for gut microbial dysbiosis in CKD patients is bacterial hydrolysis of urea by ureases within the GI tract, leading to increased gut luminal ammonia and increased intestinal pH. A 2014 study found that 63% of the 19 gut microbial families dominant in CKD patients encoded urease genes, and such bacterial communities exhibited an increase in gene products that form indoles and *p*-cresols along with a reduction in genes that produce the short chain fatty acids that are healthy for colonocytes [121]. Higher gut pH significantly induces the expression of tryptophanase, the enzyme responsible for indole production and subsequent IS formation [122]. Tryptophanase activity is also thought to limit tryptophan availability to the host which can alter serotonin levels [123,124]. Because 95% of serotonin is located in the gut, dysbiosis can impact the function of both the enteric and central nervous systems [125]. Increased intestinal pH further contributes to dysbiosis and has been shown to favor the growth of uremic toxin-forming taxa [18]. Taken together, dysbiosis-induced increases in gut-derived uremic toxins further kidney damage and exert deleterious effects on the vasculature, bone, heart, hepatic metabolism, and brain (Figure 2).

Protein digestion, metabolism, and absorption in the small intestine is also impaired in ESRD in both dialysis and non-dialysis patients [126,127]. These factors likely contribute to protein malnutrition, a common problem observed in kidney disease patients. Furthermore, proteins not metabolized or digested in the small intestine progress into the colon, where the higher density of microbial cells produces uremic toxins. Indeed, serum levels of both IS and pCS, which are influenced by diet and intestinal microbes, can be correlated with CKD disease stage and severity [128]. Using serum IS and pCS levels as diagnostic tools predictive of disease progression has been proposed [129]. Interestingly, however, a recent study showed that the levels of the IS and pCS precursors indole and *p*-cresol did not change in feces and urine as kidney disease progressed [130]. The authors proposed that while the microbial generation of these precursors may not change as disease advances, their retention and conversion into uremic toxins is caused by a progressive decline in kidney function.

Detailed alterations to the composition of the gut microflora vary between CKD patients. Dysbiosis is impacted by several variables including reduced GFR, increased colonic pH, dietary changes, pharmaceutical interventions, and other CKD-related factors [131]. These factors may work in concert to alter the biochemical milieu of the gut, colonic microbial metabolism, and the composition of the microbiota. A recent study showed that the taxa responsible for production of IS and pCS vary between kidney disease patients [132]. *Bacteroides* and *Blautia* taxa were correlated with high IS but low pCS levels in the serum, whereas *Enterococcus*, *Akkermansia*, *Dialister*, and *Ruminococcus* taxa were linked to high pCS and low IS serum levels. These findings are consistent with other reports on taxa capable of producing *p*-cresol and its precursor 4-hydroxyphenylacetate, which include *Ruminococcus* and *Veillonollaceae* [133]. In addition, this study showed that a number of *Bacteroides* taxa metabolized all three aromatic amino acids to produce *p*-cresol, IAA, and phenylacetic acid. Indeed, Devlin et al. revealed that nearly 40% of tryptophanases present in the gut are expressed by *Bacteroides* [134]. However, many *Bacteroides* species native to the intestine, including *B. fragilis*, *B. vulgatus*, and *B. caccae*, do not express this indole-producing enzyme. Numerous toxin-producing bacteria were shown to be enriched in ESRD patients compared to controls and were correlated with patient clinical parameters [135]. *Eggerthella lenta* and *Fusobacterium nucleatum* were the most enriched species in ESRD patients and both taxa play a role in the production of gut-derived PBUTs and their precursors like indole, phenol, and HA. Lastly, Kim et al. correlated serum levels of IS, pCS, and *p*-cresyl glucuronide to alterations in the gut microbiota of 103 CKD patients with mild, moderate, and severe disease [136]. The authors found that *Alistipes* and *Oscillobacter* taxa were correlated with IS and *p*-cresyl glucuronide levels, and *Alistipes*, *Oscillobacter*, and *Subdoligranulum* taxa correlated with pCS levels. *Oscillobacter* was suggested to act as a hub in the microbial networks of patients with moderate and severe disease, with its abundance giving rise to other CKD-associated taxa. A deeper understanding of the driver and passenger bacterial species in CKD that express enzymes responsible for PBUT production may lead to targeted treatments to reduce serum accumulation of toxins like IS and pCS.

Alterations in the chemical environment of the gut due to CKD can also impact neuroendocrine pathways, including the hypothalamus–pituitary–adrenal axis (HPA axis) and the production of neurotransmitters and neuroactive compounds [137]. The HPA axis is activated in response to stress and stimulates the central nervous system [138]. Because toxic compounds like bacterial peptidoglycans and endotoxins more readily cross the gut endothelial barrier due to the increased permeability associated with CKD, they subsequently stimulate the HPA axis to induce a stress response [139,140]. The short chain fatty acids propionate and butyrate produced by gut microbes alter the expression of peptide-YY, an important regulator of food intake and insulin secretion [141]. Dysbiosis in CKD decreases the expression of genes that are responsible for the production of short chain fatty acids, leading to alterations in peptide-YY levels and impacting the pathophysiology of obesity and diabetes, important risk factors of CKD. Finally, the gut microbiota influences the production of a number of neurotransmitters and neuroactive compounds, including GABA, serotonin, tryptamine, catecholamine, and acetylcholine [142], that together impact homeostasis and blood pressure, factors that significantly affect CKD and cardiovascular disease progression.

### 3.2. Reducing Gut-Derived PBUTs

Probiotics, prebiotics, and synbiotics have been explored in preclinical and clinical studies as therapeutic strategies for CKD. Their impacts on CKD are measured using gut-derived uremic toxin levels, inflammatory markers, and blood urea nitrogen levels. Results for probiotics are conflicting, with some studies showing their ability to reduce IS and pCS levels [143,144], while others found no beneficial effects [145]. Investigations of prebiotics, food ingredients directed to the microbiota [146], have shown that such compounds caused Stage 3–5 CKD patients to exhibit a reduction in plasma levels of gut-derived uremic toxins, particularly IS and pCS [147,148]. Such interventions are thought to favor the growth of healthy gut microbes that restore barrier function while decreasing levels of bacteria that produce uremic toxins [149]. The use of synbiotics, combinations of pre- and probiotics, in animal models and CKD patients reduces blood urea nitrogen, inflammatory markers, and gut-derived uremic toxin levels [150,151,152].

Diet directly impacts the composition and activity of the gut microbiota. A very low protein diet (0.3 g/kg/day of protein) supplemented with ketoanalogues like ketoleucine and ketoalanine reduced IS serum levels in CKD patients by 37% after only 1 week [153]. Furthermore, 6 months of a low-protein diet in non-dialyzed CKD patients produced a marked decrease in serum pCS levels and favorable changes to the gut microbiota composition [154]. Numerous studies confirm that high protein intake, especially in the form of red meat, increases the production of the gut-derived uremic toxins IS, indoxyl glucuronide, kynurenic acid, quinolinic acid, and pCS [155,156]. Therefore, lowered protein intake and vegetarian diets will likely reduce gut-derived uremic toxin levels. Diets with a high protein-to-carbohydrate ratio favor the prevalence of proteolytic bacteria that produce uremic toxins over saccharolytic bacteria that generate beneficial short chain fatty acids. In contrast, a diet that is high in carbohydrates and whole grain fibers but low in red meat, such as a Mediterranean diet, promotes the growth of saccharolytic taxa that reduce gut-derived uremic toxin levels [157,158].

Integrating a low-protein diet with synbiotic supplementation is a promising tool to correct protein assimilation, control disease progression, and improve gut intestinal barrier integrity. Validation of this approach in a large-scale clinical trial is required to demonstrate reduced uremic toxin levels and better outcomes in CKD patients. Low patient adherence is a serious caveat when prescribing dietary alterations and supplementation, especially in disease states with risk factors such as diabetes, obesity, and cardiovascular disease.

The use of carbon adsorbents and phosphate binders to reduce gut-derived uremic toxins is another strategy employed in CKD. AST-120, a spherical carbon adsorbent, was designed to sequester toxins within the GI tract, reducing their absorption and subsequent accumulation in the serum. A number of preclinical and randomized controlled studies have tested this strategy and results are mixed, with clinical and post-hoc analyses showing toxin reductions [159,160,161] and others, particularly the primary randomized controlled trial, showing no proof of utility for CKD [162,163]. Regardless, AST-120 has been approved for CKD treatment in Korea, Taiwan, and the Philippines, and is believed to prolong the time to the initiation of dialysis [161]. Phosphate binders like sevelamer and nicotinamide that sequester phosphate in the GI tract, are believed to improve inflammatory status and may enhance the clearance of uremic toxins. Indeed, studies establish that sevelamer, but not nicotinamide, reduces pCS serum levels but did not impact IS concentrations [164,165,166].

Lubiprostone, an FDA approved ClC-2 chloride channel activator used to treat constipation in patients with irritable bowel syndrome [167], was studied in adenine-induced renal failure mouse models. This bicyclic fatty acid derivative improved fecal and intestinal properties of the animals, promoted the recovery of *Lactobacillaceae* and *Prevotella* taxa, and reduced serum levels of IS and HA [168]. Lubiprostone has not been studied in CKD patients to date. For additional information on the relationship between the gut microbiota and CKD, the reader is directed to Plata et al. [169] and Castillo-Rodriguez et al. [170].

## 4. Conclusions

Gut-derived, protein-bound uremic toxins have a range of deleterious effects, including altered hepatic metabolism, advancement of renal fibrosis, induction of atherosclerosis, and neurotoxicity. Each class of toxin has a unique derivation pathway and pathological mechanism, but they can ultimately be separated into diet-derived or microbial metabolism-derived categories. Gut dysbiosis in chronic kidney disease not only causes the increased production of uremic toxins but also negatively affects the immune system and intestinal permeability. Furthermore, because some of the most damaging uremic toxins derive from the gut, modification of the intestinal microflora represents a promising therapeutic strategy. Current interventions aimed at preventing the serum accumulation of gut-derived uremic toxins have focused on dietary restrictions, synbiotics, and oral adsorbents, and all are moderately effective. Further strategies for reducing protein-bound uremic toxins are the addition of sorbents and binding competitors to dialysate, as well as the use of super-flux and coated membranes in dialysis [171]. Although promising, these alterations to dialysis techniques fail to address gut dysbiosis and are only available to patients that have already progressed into ESRD. As such, most kidney disease patients lack an effective therapeutic strategy to combat uremic syndrome. While 500 million people suffer from CKD globally, there are fewer interventional trials for therapeutics for kidney disease than almost any other medical subspecialty [172]. The development of novel therapeutics that target the gut microbiota may correct the underlying factors that drive uremic syndrome, gut dysbiosis, and CKD.

## Figures and Tables

**Table 1:** Gut-Derived Protein-Bound Uremic Toxins.

Gut-Derived PBUT Class	Toxin	Derivation	Pathological Mechanisms	Associated Comorbidities
AGEs	3-Deoxyglucosone Fructoselysine Glyoxal Methylglyoxal N(6)-Carboxymethyllysine Pentosidine	Diet	ECM crosslink formation Impaired endothelial progenitor cell function NF- kB/MAPK/JNK signaling RAGE signaling	Arterial stiffness Diabetic nephropathy Endothelial dysfunction Immune system dysregulation
Hippurates	Hippuric acid Hydroxyhippuric acid	Diet	Activation of mitochondrial fission Albumin binding Free radical production NF- kB signaling	Altered drug pharmacokinetics Endothelial dysfunction Renal tubule damage
Indoles	Indole-3-acetic acid Indoxyl glucuronide Indoxyl sulfate Kynurenine Kynurenic acid Melatonin Quinolinic acid	Microbial metabolism	AhR activation Excessive glutamate release Impaired mitochondrial OXPHOS NF- kB/MAPK signaling NMDA receptor activation Reduced PTH expression	Bone disease Cardiovascular disease Endothelial dysfunction Inflammation Muscle weakness/atrophy Neurotoxicity Oxidative stress
Phenols	Hydroquinone *p*-cresyl glucuronide *p*-cresyl sulfate Phenol Phenylacetic acid	Microbial metabolism	Apoptosis Chromosomal aberrations Inhibition of iNOS expression NADPH oxidase activation ROS production Stimulates Rho-associated protein kinase	All-cause mortality Cardiovascular disease Inflammation Oxidative stress Renal fibrosis Vascular remodeling
Polyamines	Putrescine Spermidine Spermine	Microbial metabolism/Diet	Inhibition of erythropoietin	Anemia
Other	CMPF Homocysteine	Diet	Albumin binding Altered hepatic metabolism CMPF radical adducts Competitive reabsorption by OAT Degradation of gut epithelial TJ VSMC proliferation	Altered drug pharmacokinetics Atherosclerosis Increased intestinal permeability Neurological abnormalities Renal tubule damage

AGE, advanced glycation end product; AhR, aryl hydrocarbon receptor; CMPF, 3-carboxy-4-methyl-5-propyl-2-furanpropionic acid; ECM, extracellular matrix; iNOS, nitric oxide synthase; JNK, c-Jun N-terminal kinase; MAPK, mitogen-activated protein kinase; NF- kB, nuclear factor kappa B; NMDA, N-methyl-D-aspartate; OAT, organic anion transporter; OXPHOS, oxidative phosphorylation; PBUT, protein-bound uremic toxin; PTH, parathyroid hormone; RAGE, advanced glycan end product-specific receptor; ROS, reactive oxygen species; TJ, tight junctions; VSMC, vascular smooth muscle cell.
